# Investigating the application of “Guttmann Cognitest”^®^ in older adults and people with acquired brain injury

**DOI:** 10.3389/fneur.2023.1292960

**Published:** 2024-01-08

**Authors:** Gabriele Cattaneo, Alba Roca-Ventura, Eva Heras, Maria Anglada, Jan Missé, Encarnació Ulloa, Simon Fankhauser, Eloy Opisso, Alberto García-Molina, Javier Solana-Sánchez

**Affiliations:** ^1^Institut Guttmann, Institut Universitari de Neurorehabilitació Adscrit a la UAB, Badalona, Spain; ^2^Departament de Medicina, Universitat Autònoma de Barcelona, Bellaterra, Spain; ^3^Fundació Institut d'Investigació en Ciències de la Salut Germans Trias i Pujol, Barcelona, Spain; ^4^Departament de Medicina, Facultat de Medicina i Ciències de la Salut, Universitat de Barcelona, Barcelona, Spain; ^5^Servei Envelliment i Salut Servei Andorrà d'Atenció Sanitària, Andorra la Vella, Andorra

**Keywords:** aging, cognitive decline, cognitive functioning, computerized cognitive assessment, memory

## Abstract

**Introduction:**

Digital solutions for cognitive assessment are currently not only widely used in experimental contexts but can also be useful in clinical practice for efficient screening and longitudinal follow-up. The “Guttmann Cognitest”^®^, which includes seven computerized tasks designed to assess main cognitive functions, revealed in a previous validation study to be a potential useful tool to assess cognitive functioning in healthy middle-aged adults.

**Method:**

Here, we present results from a validation in two different populations: one consisting of older adults, and the other comprising young and middle-aged individuals, some of them affected by acquired brain injury. To perform a convergent validity test, older adults were also administered with the MOCA, while young and middle-aged individuals were administered with a short neuropsychological assessment including gold-standard neuropsychological tests. We also conducted sensitivity and specificity analysis to establish the utility of this instrument in identifying potential cognitive dysfunctions in the two groups.

**Results:**

Results demonstrated strong convergent validity as well as good specificity and sensitivity characteristics.

**Discussion:**

This tool is a valid and useful instrument to assess cognitive functioning and detecting potential cases of cognitive dysfunctions in older adults and clinical populations.

## 1 Introduction

As life expectancy continues to rise, healthcare services around the world will be progressively and increasingly challenged by age-related diseases (1). It has been estimated that the proportion of people over 55 years will surpass people under 15 years by the year of 2035 (2). Advancing age represents the main risk factor for the development of brain related diseases (3).

For example, Alzheimer's disease and other forms of dementias is the 7th causes of death worldwide, the second in high income countries (4, 5). Estimations on disability further propose that in 2050, half of the global burden due to disability will be attributable to mental and cognitive disorders (6). Additionally, aging itself is recognized to lead to the decline or alteration of structural and functional brain mechanisms (7), resulting in subsequent functional impact.

In this context early detection of cognitive decline is crucial to implement preventive strategies that can halt the progression of decline and promote brain health maintenance (8, 9). To achieve this aim there is the need to implement efficient, and accessible cognitive assessments that can allow us to identify preclinical stages of diseases and detect subtle cognitive changes over time. This is especially relevant for Alzheimer's disease and other neurodegenerative pathologies preceded by a long preclinical phase. The early detection of these cases could have significant implications for individuals' quality of life and level of independence afterward. Once detected and diagnosed, these conditions could be accompanied by an appropriate and efficient follow-up for monitor cognitive changes and evaluate the potential effects of prescribed interventions.

Unfortunately, the current reality is that Alzheimer's disease is largely underdiagnosed (10). This underdiagnosis is even more pronounced for preclinical subtle symptoms due to limitations in primary care, such as a lack of specially qualified personnel, resources, and optimal tools. Recent data showed that even if 96% of physicians express the desire to screen for cognitive functioning, 50% of older adults undergo effective examinations, and only 15% periodically (11).

Addressing these challenges and improving the rates of detection and follow-up is essential to ensure timely intervention and support for individuals at risk of cognitive decline. By enhancing access to appropriate assessment tools, it will become possible to increase the identification of cognitive changes at an early stage and enable the implementation of preventive measures. It has been estimated that delaying the onset of symptoms of dementia of only 1 year could prevent in over 11.8 million cases in the next 30 years, reducing expenses for healthcare of $219 billion (12, 13).

In this scenario, mobile technologies and computerized cognitive assessment may represent the solution (14).

This kind of tools have been developed and studied for years, showing to be a usable and reliable solution. They offer the potential to perform population based longitudinal screening that will allow to early detect cognitive changes, without incurring the costs associated with administering in-person neuropsychological tests (11).

Following the same logic, these tools could be extremely useful also in clinical contexts to monitor people diagnosed with brain pathologies, as acquired brain injuries (ABI) (15). It could allow to optimize human and economic resources and improve healthcare quality by simplifying and speeding up patient's assessments, and maximizing patient's monitoring possibilities, as done for non-cognitive outcomes [see for example (16)].

However, in the specific contexts of people with ABI the use of this digital solution could be complicated by patient's characteristics and heterogeneity. Concretely, the usability of these solutions could be potentially reduced due to motor, visual, comprehension, or other pathology's related impairments that difficult or totally prevent tasks execution. In these cases, the administration would probably need to be filtered and guided by a health care professional.

In a previous study, we explored the validity and usability of the “Guttman Cognitest,” a digital solution to assess cognitive functioning, in middle aged and cognitively unimpaired adults (17) and calculated regression-based norms.

Here, we aim to extend previous findings further exploring convergent validity. Moreover, we studied the capacity of this tool to detect possible cases of cognitive impairment.

## 2 Materials and methods

### 2.1 Participants

A total of 318 young and middle-aged adults (141 women; mean age = 54.5, range = 19–69) and 71 older adults over 70 years (43 women; mean age = 76.7, range = 70–88) participated in this study (see Table 1).

**Table 1:** Sociodemographic characteristics of participants.

	**Variable**	**Mean (SD)**	**Percentage (%)**
Older adults	Age	76.7 (5.1)	-
	Sex	-	Female 61.6
	Education level	-	Primary: 47.9
			Secundary: 43.7
			Superiors: 8.4
Young and middle-aged adults	Age	54.4 (9.1)	-
	Sex	-	Female 44.3
	Education level	-	Primary: 8.2
			Secundary: 30.2
			Superiors: 61.6

The group of older adults were composed of people recruited from Andorra and participating in the Integrated care for older people (ICOPE) program promoted by the WHO. This program is part of a population-based study conducted by the Andorran Health Care System with the aim of identifying population frailty. In this group 39 subjects presented MCI according with classically used clinical criteria [MOCA < 26; (18–20)], while 32 participants scored 26 or more.

Young and middle-aged adults instead were patients of the Neurorehabilitation Hospital Institut Guttmann, and people participating in the Barcelona Brain Health Initiative (8).

In this group 44 participant were affected by ABI (See Table 2 for etiology of brain injuries), and between them 42 resulted cognitively impaired in at least one cognitive function.

**Table 2:** Etiology of acquired brain injury in patients of Neurorehabilitation Hospital Institut Guttmann.

**Etiology of brain injuries**	***N***	**Percentage (%)**
Traumatic brain injury	17	38.6
Hemorrhagic stroke	10	22.7
Ischemic stroke	8	18.2
Multiple Sclerosis	3	6.8
Anoxia	3	6.8
Guillain-Barre syndrome	2	4.6
Brain tumor	1	2.3

All participants provided explicit informed consent, and the protocol was approved by the Ethics and Clinical Research Committee of the Catalan Hospitals Union (Comité d'Ètica I Investigació Clínica de la Unió Catalana hospitals).

Participants with motor and visual impairments, as well as comprehension deficits that could alter tasks execution were excluded from this study.

### 2.2 Procedures

Older adults were administered with the “Guttmann Cognitest”^®^ and the Montreal Cognitive Assessment (MOCA) (21) on the same day. On the other hand, young and middle-aged adults were administered with the “Guttmann Cognitest”^®^ and a neuropsychological test battery, by expert's neuropsychologist, on different days (mean difference between tests administration in days = 4.1; see Table 3 for results). The administration of the digital solution in people with ABI and older adults was supervised by an expert neuropsychologist to assure that they did not present visual, physical and comprehension impairments that could alter their results.

**Table 3:** Results of the neuropsychological tests for older adults and people with ABI.

**Grup**	**Nuropsychological test**	**Mean (SD*)***
Older adults	MOCA	23.9 (4.6)
Acquired brain injury	TMT A	32.5 (26.8)
	Digit Span Forward	5.6 (1.4)
	RAVLT Immediate Recall	52.8 (12.1)
	RAVLT Delayed Recall	11.4 (3.6)
	RAVLT Recognizing	13.9 (2.4)

The neuropsychological assessment was designed to measure visuo-spatial searching, and attention [Trail making test A; (22)], working memory [Digit Span forward; (23)] and episodic memory [Rey Auditory Verbal Learning Test (RAVLT) (24)].

The “Guttmann Cognitest”^®^ includes 7 tasks designed to assess memory, executive functions, and visuo-spatial abilities [see (17) for a detailed description of the tasks].

All tasks are preceded by a set of screens with detailed instructions together with a video tutorial, explaining the objective of the task and the expected behavior of the user.

After this, a simple demo screen of the task plays as practice for the user, to ensure that they have understood the logic, objectives and expected responses of the task. Only once the practice is completed correctly (there are two attempts) the corresponding task begins. If the practice is not completed correctly the task is not administered and the system moves directly to the next task, assuming that the person would not have been able to complete that task correctly due to task comprehension.

### 2.3 Data and statistical analysis

Following the same procedure as reported in previous studies [e.g., (17, 25–27)], we transformed raw scores obtained in gold-standard neuropsychological tests into z-scores and then calculated a global-cognition score as their mean.

Z-scores for the “Guttmann Cognitest”^®^ were calculated using the formulae estimates with the regression-based norms previously published (17), and global cognition composite score was determined by taking the mean of all the transformed z-scores. As typical norms for neuropsychological classical tests these formulae correct the raw score for age, biological sex and educational level to control for their effects on the results.

Convergent validity was assessed using Spearman's rank correlations between the global composite score of the “Guttmann Cognitest”^®^ and results obtained in the classical neuropsychological tests (MOCA for older adults, and global composite score for young and middle-aged adults).

We then performed a receiver operating characteristic curve in both groups (ROC curve) to estimate the value that showed the best sensitivity and specificity characteristics to detect cognitive impairments. This was defined as a score inferior to 26 at the MOCA (18–20), or people with ABI that scored below the cut-off usually employed for clinical practice, and based on Spanish normative data, in at least one of the classical neuropsychological tests (42 out of 44 patients).

All statistical analyses were performed in R version 4.2.1 (28), and run in RStudio, version 2023.06.1 (29).

## 3 Results

### 3.1 Convergent validity

#### 3.1.1 Older adults

**Figure 1:**
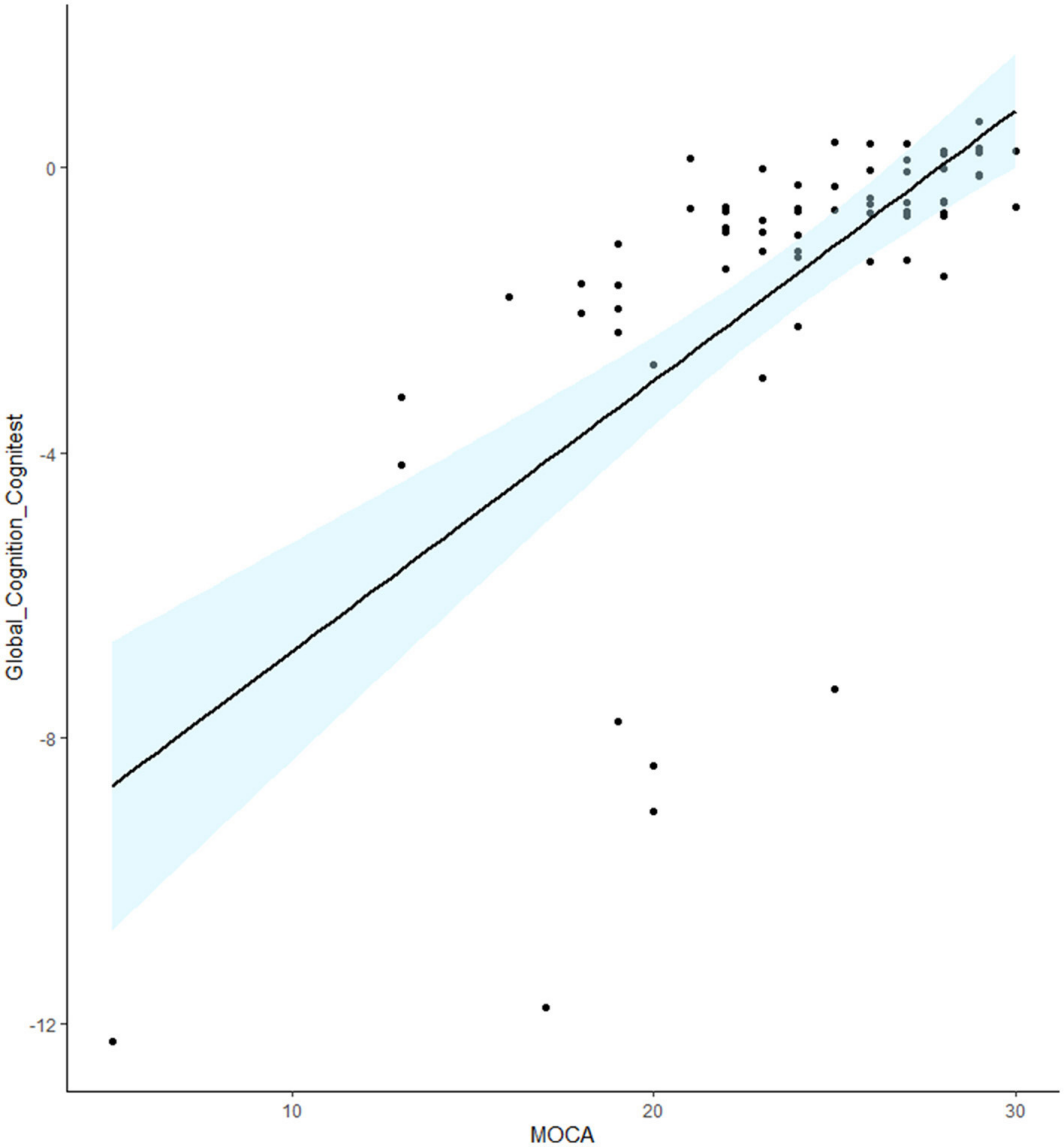
Scatterplot representing the correlation between MOCA scores and global cognition composite scores obtained with the Cognitest.

The Global “Cognitest” composite score showed strong correlation with the MOCA score (rs = 0.71; *p* < 0.001) indicating good convergent validity (Figure 1).

#### 3.1.2 Young and middle-aged adults

**Figure 2:**
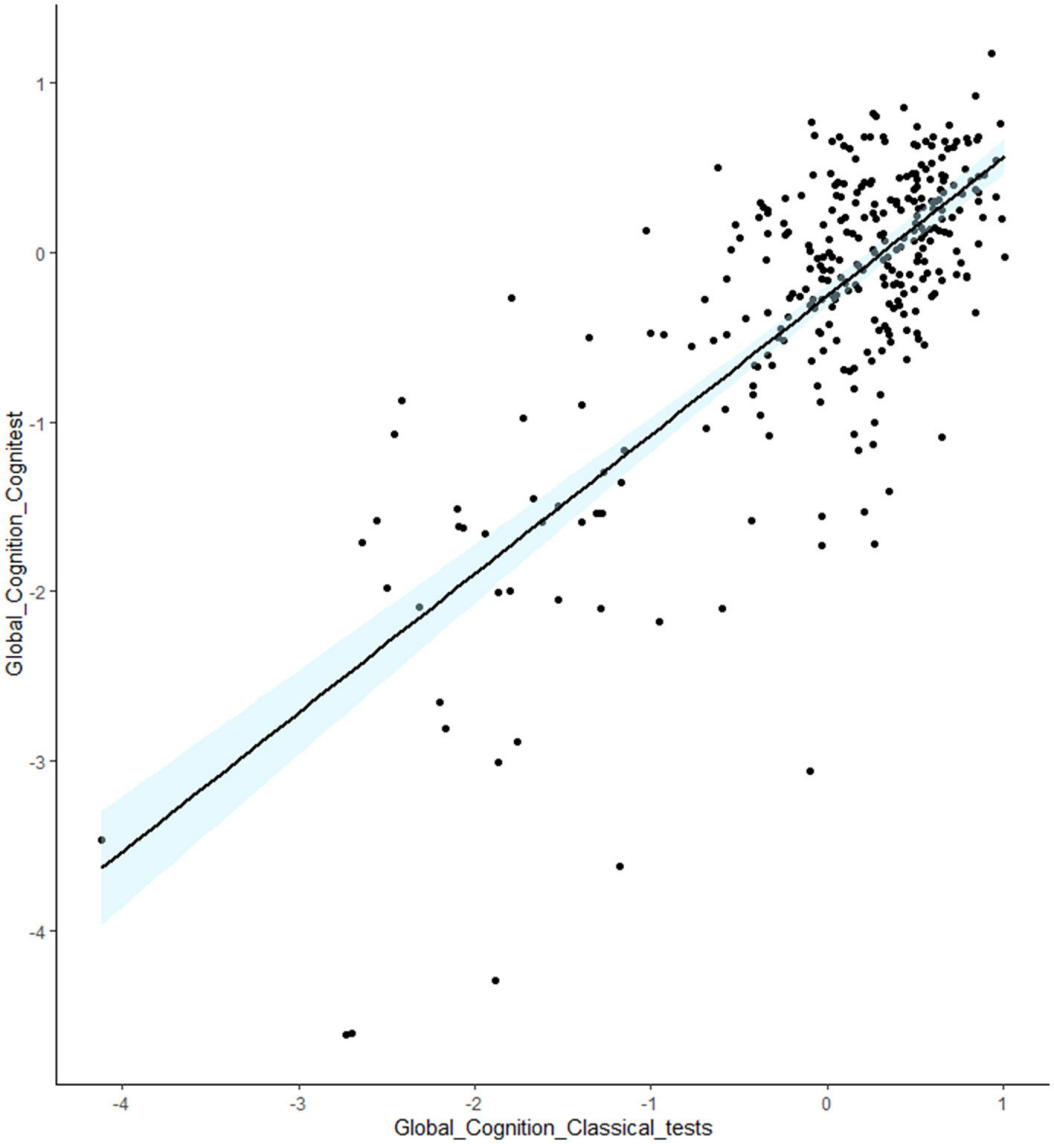
Scatterplot representing the correlation between global cognition scores obtained with the standard tests, and global cognition composite scores obtained with the Cognitest.

Also, in this group we found a strong correlation between Global Cognitest composite score, and Global composite score calculated for classical paper and pencil neuropsychological tests (rs = 0.60; *p* < 0.001), confirming good convergent validity (Figure 2).

### 3.2 Receiver operating characteristic curve

#### 3.2.1 Older adults

**Figure 3:**
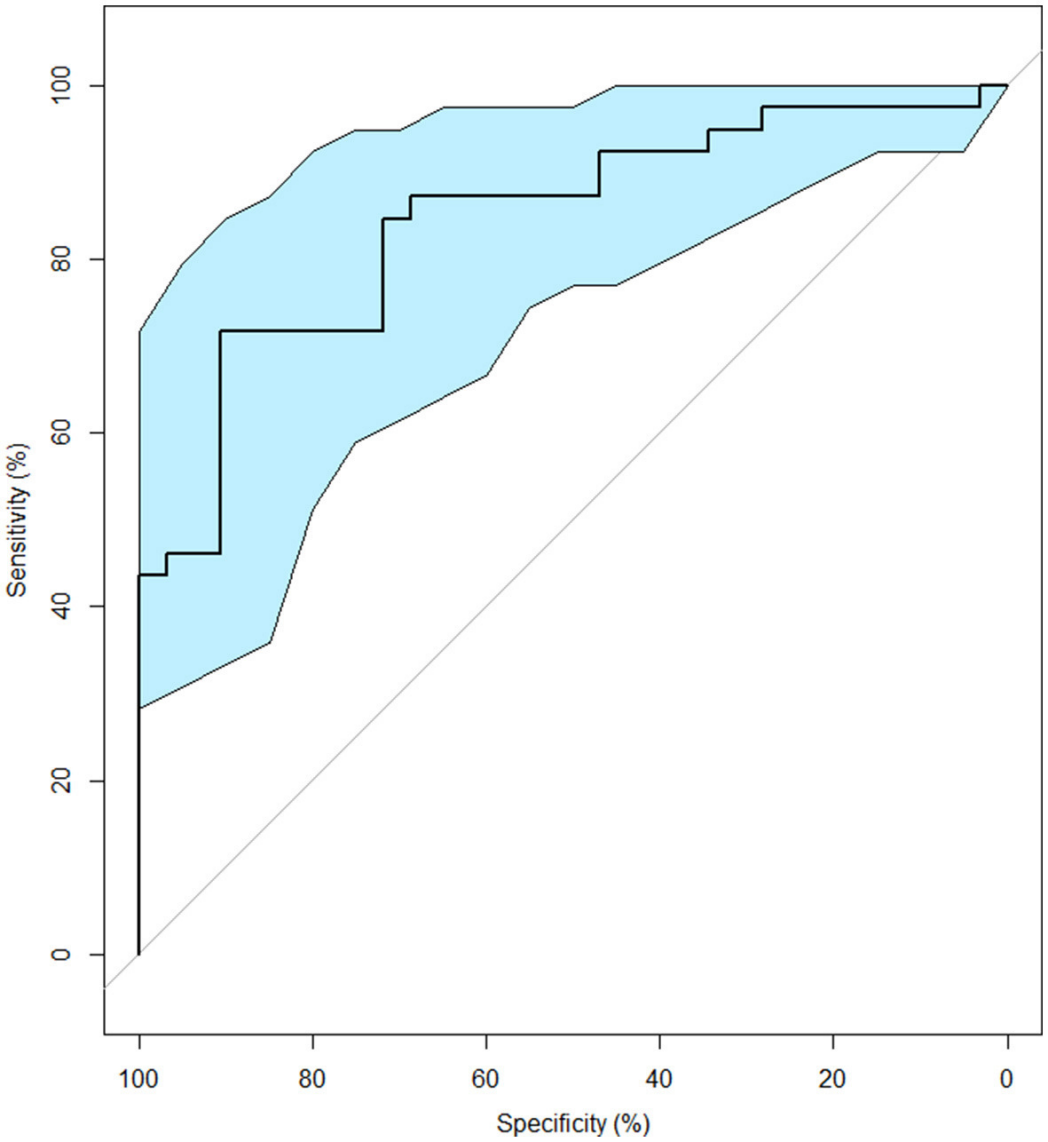
ROC curve for detecting MCI in older adults. The light blue shape represents the CI 95%.

The analysis revealed an area under the curve of 0.84 (CI 95%: 0.75–0.93), indicating a satisfactory discriminant capacity. According to Youden's J statistics, the optimal discriminant Global Cognitest z-score value was found to be−0.70, resulting in a sensitivity of 0.72 and a specificity of 0.91 (Youden's index = 0.62; see Figure 3).

This cut-off value correctly identified 28 out of 39 individuals presenting MCI and only resulted in 3 false positive out of 32 cases.

#### 3.2.2 Young and middle-aged adults

**Figure 4:**
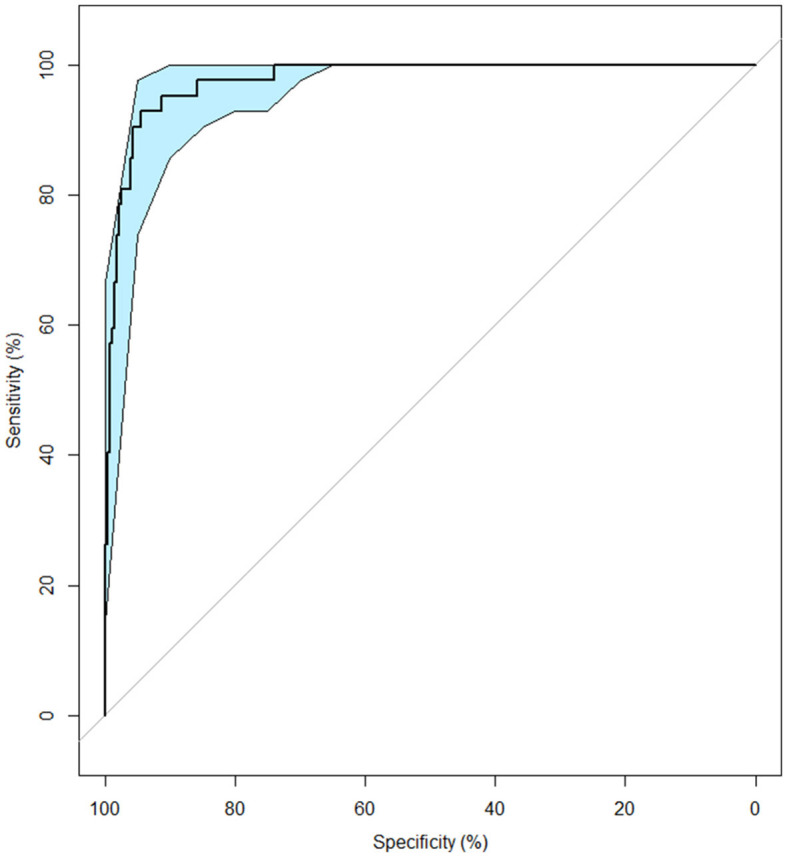
ROC curve for detecting cognitive impairments due to ABI in young and middle-aged adults. The light blue shape represents the CI 95%.

The area under the curve was 0.98 (CI 95%: 0.96–0.99), indicating a very good discriminant capacity. With a z-score of −0.85, sensitivity was 0.93 and a specificity 0.95 (Youden's index = 0.87; see Figure 4).

This cut-off value correctly identified 39 out of 42 individuals presenting cognitive impairments and produced only 15 false positive out of 276 unimpaired individuals.

## 4 Discussion

In this study, we aimed to explore convergent validity between global cognitive performance measured by classical neuropsychological tests, and by the Guttmann Cognitest digital solution in older adults and a group of young and middle adults. Moreover, we studied its utility in detecting in these groups, respectively possible cases of MCI and cognitive dysfunctions in people presenting ABI.

Results showed strong correlation between a global score obtained on the Cognitest and global cognitive functioning calculated with classical paper and pencil neuropsychological tests. This indicates a very good convergent validity in both older adults and young and middle-aged adults.

Present findings are in line with previous validation study of computerized neuropsychological assessment tools in healthy adults and clinical populations [see (11, 15, 30)], and our previous validation (17). These studies demonstrated some variability in terms of convergent and concurrent validity, ranging from small to large effect sizes (from 0.2 to 0.88).

The variability observed in these results makes it somewhat challenging to establish clear expectations regarding the desired effect sizes for an effective solution.

However, considering the results obtained, the Guttmann Cognitest exhibited overall a good convergent validity when compared to established neuropsychological tests considered as the gold standard.

Crucially, the receiver operating characteristic analysis showed a specificity and sensitivity characteristics in line with those of other classical screening tests used to detect cognitive impairments and MCI [see (31–33) for review].

This result demonstrate that this digital solution represent a potentially useful tool to be employed in clinical populations to identify mild cognitive impairment (34) and other cognitive dysfunctions.

The present study presented some limitations that future validation studies must consider and solve. We did not screen participants for depression, that could have biased their performance at the tests. Moreover, two of the three memory tasks (Cued image-number association and long term memory) showed significant ceiling effects, showing to be too easy, and with potential reduced discriminant capacity (See Supplementary material for details). However, if we consider the whole tasks, as for calculating “Global cognition” composite scores, the ceiling effect is reduced.

Taken together, these results indicate that digital solutions, such as ours, have the potential to serve as a benchmark for conducting large-scale population screening, and a useful tool for clinics to increase healthcare quality. Indeed, this can effectively reduce the underdiagnosis of cognitive impairment and potentially detect preclinical cognitive changes in aging, as well as help for patient's cognitive screening.

Together with its potential to efficiently monitor cognition over time, these innovative procedures will allow to improve patient's follow-up and implement preventive strategies. This can reduce the progression of cognitive decline and promote resilience, having an important impact on people's health and wellbeing, and an enormous economic impact on society.

In conclusion, our findings demonstrate the utility of digital solutions like the “Guttmann Cognitest”^®^ as a reliable and efficient tool for evaluating cognitive functions in older adults and clinical populations.

## Data availability statement

The raw data supporting the conclusions of this article will be made available by the authors, without undue reservation.

## Ethics statement

The studies involving humans were approved by the Comite d'Etica de la Unió Catalana d'Hospitals. The studies were conducted in accordance with the local legislation and institutional requirements. The participants provided their written informed consent to participate in this study.

## Author contributions

GC: Conceptualization, Data curation, Formal analysis, Investigation, Methodology, Writing—original draft. AR-V: Conceptualization, Investigation, Writing—review & editing. EH: Conceptualization, Investigation, Supervision, Writing—review & editing. MA: Data curation, Investigation, Writing—review & editing. JM: Investigation, Writing—review & editing. EU: Investigation, Writing—review & editing. SF: Investigation, Writing—review & editing. EO: Conceptualization, Investigation, Supervision, Writing—review & editing. AG-M: Conceptualization, Investigation, Supervision, Writing—review & editing. JS-S: Conceptualization, Investigation, Supervision, Writing—review & editing.
