# Supplementary material for: Investigating the application of “Guttmann Cognitest”® in older adults and people with acquired brain injury
Source: Front Neurol. 2024 Jan 8;14:1292960. doi: 10.3389/fneur.2023.1292960 (PMC10800697; doi:10.3389/fneur.2023.1292960)
Supplement: Supplementary file 1 [file Table_1.pdf]

Supplementary material

Table 1. Floor and ceiling effect of the tasks included in the Cognitest. Number represents the proportion of individuals scoring the highest (ceiling) or lowest (floor) possible score across any given task (Gulledge et al., 2019).

Task	% of Floor effects	% of Ceiling effects
Visual span backward	2.7	0
Free image-number association	5.7	0.8
Cued image-number association	1.0	27.1
Long term memory	0	51.9
Logic sequences	1.1	7.3
Cancellation	0	0
Circle tapping accuracy	0	0
Circle tapping reaction times	0	0
Mental rotation	0.3	2.9
